# The Multifunctional LigB Adhesin Binds Homeostatic Proteins with Potential Roles in Cutaneous Infection by Pathogenic *Leptospira interrogans*

**DOI:** 10.1371/journal.pone.0016879

**Published:** 2011-02-09

**Authors:** Henry A. Choy, Melissa M. Kelley, Julio Croda, James Matsunaga, Jane T. Babbitt, Albert I. Ko, Mathieu Picardeau, David A. Haake

**Affiliations:** 1 Department of Medicine, David Geffen School of Medicine at University of California Los Angeles (UCLA), Los Angeles, California, United States of America; 2 Research Service, Veterans Affairs Greater Los Angeles Healthcare System, Los Angeles, California, United States of America; 3 Goncalo Moniz Research Center, Oswaldo Cruz Foundation, Brasilian Ministry of Health, Salvador, Bahia, Brasil; 4 Faculty of Health Sciences, Federal University of Grande Dourados, Dourados, Mato Grosso do Sul, Brasil; 5 Division of Epidemiology of Microbial Diseases, Department of Epidemiology and Public Health, Yale University School of Medicine, New Haven, Connecticut, United States of America; 6 Institut Pasteur, Unite de Biologie des Spirochetes, Paris, France; 7 Division of Infectious Diseases, Veterans Affairs Greater Los Angeles Healthcare System, Los Angeles, California, United States of America; 8 Department of Urology and Department of Molecular Genetics, Microbiology, and Immunology, University of California Los Angeles, Los Angeles, California, United States of America; Instituto Butantan, Brazil

## Abstract

Leptospirosis is a potentially fatal zoonotic disease in humans and animals caused by pathogenic spirochetes, such as *Leptospira interrogans*. The mode of transmission is commonly limited to the exposure of mucous membrane or damaged skin to water contaminated by leptospires shed in the urine of carriers, such as rats. Infection occurs during seasonal flooding of impoverished tropical urban habitats with large rat populations, but also during recreational activity in open water, suggesting it is very efficient. LigA and LigB are surface localized proteins in pathogenic *Leptospira* strains with properties that could facilitate the infection of damaged skin. Their expression is rapidly induced by the increase in osmolarity encountered by leptospires upon transition from water to host. In addition, the immunoglobulin-like repeats of the Lig proteins bind proteins that mediate attachment to host tissue, such as fibronectin, fibrinogen, collagens, laminin, and elastin, some of which are important in cutaneous wound healing and repair. Hemostasis is critical in a fresh injury, where fibrinogen from damaged vasculature mediates coagulation. We show that fibrinogen binding by recombinant LigB inhibits fibrin formation, which could aid leptospiral entry into the circulation, dissemination, and further infection by impairing healing. LigB also binds fibroblast fibronectin and type III collagen, two proteins prevalent in wound repair, thus potentially enhancing leptospiral adhesion to skin openings. LigA or LigB expression by transformation of a nonpathogenic saprophyte, *L. biflexa*, enhances bacterial adhesion to fibrinogen. Our results suggest that by binding homeostatic proteins found in cutaneous wounds, LigB could facilitate leptospirosis transmission. Both fibronectin and fibrinogen binding have been mapped to an overlapping domain in LigB comprising repeats 9–11, with repeat 11 possibly enhancing binding by a conformational effect. Leptospirosis patient antibodies react with the LigB domain, suggesting applications in diagnosis and vaccines that are currently limited by the strain-specific leptospiral lipopolysaccharide coats.

## Introduction

Leptospirosis is a potentially fatal zoonotic disease in humans and animals caused by pathogenic spirochetes, such as *Leptospira interrogans* [1], [2], [3]. The mode of transmission is contact with environmental water contaminated by leptospires shed in the urine of carriers, such as rats. Leptospirosis is normally neither communicable in humans nor transmitted by animal bite, and the route of infection is commonly limited to damaged skin or exposed mucous membrane. Thus, the wide range of its occurrence from exposure during seasonal flooding of impoverished tropical urban habitats with large rat populations to recreational activity in open water attests to the efficiency of leptospiral infection [4], [5], [6], [7]. The initial attempts to attach to a skin injury and disseminate into the host occur as the pathogen encounters the wound healing process as well as immune responses of the host. Fibronectin and fibrinogen are major homeostatic proteins involved in the critical process of hemostasis in a fresh wound and in subsequent tissue repair [8], [9], [10], [11]. In addition, fibroblasts that newly populate an injury deposit cellular fibronectin and collagen type III to rebuild extracellular matrix [8], [9], [10], [11], [12], [13]. Fibronectin and fibrinogen are also major constituents in the circulation where they could potentially interact with the leptospires in their hematogenous dissemination to distal tissue. Both plasma fibronectin and fibrinogen can also be deposited in extracellular matrix or become associated with host cells. We previously showed that both host proteins are indeed ligands for LigA and LigB, two outer membrane proteins in pathogenic *Leptospira* strains that are members of a superfamily of bacterial proteins containing immunoglobulin-like repeats with adhesive properties [14], [15]. LigA and LigB with 13 and 12 immunoglobulin-like repeats, respectively, are highly and rapidly inducible in pathogenic *Leptopira interrogans* grown under conditions mimicking the physiological osmolarity of bodily fluids [15], [16], [17]. Moreover, the osmotic induction of Lig enhances binding to fibronectin and fibrinogen along with other host proteins in an *in vitro* model of leptospiral adhesion to extracellular matrix [15]. Recombinant LigB has higher affinity than LigA for host proteins [15], and the current study focuses on the potential role of the former during cutaneous infection early in leptospirosis.

We now show that LigB binds fibroblast-derived fibronectin with high avidity along with collagen type III, which is consistent with leptospiral attachment to a wound being mediated by LigB. In order to further demonstrate the importance of Lig as a leptospiral adhesin, we show that the genetic transformation of nonpathogenic *L. biflexa* with *ligA* or *ligB* from *L. interrogans* increases the adherence of the surrogate to immobilized fibronectin (manuscript submitted by C. P. Figueira, J. Croda, H. A. Choy, *et al.*) and fibrinogen. Studies by others have shown that LigB binds elastin, too [18], which is abundant in skin and accessible in damaged vasculature. In addition to being an adhesin, LigB disrupts the thrombin-mediated clotting cascade, suggesting that Lig-fibrinogen binding could affect hemostasis and possibly impair wound healing, thereby permitting leptospiral entry into the circulation, dissemination, as well as further infection. Taken together, the results showing LigB binding to homeostatic proteins prevalent in wounds and also inhibiting fibrin formation suggest that LigB could facilitate leptospiral transmission and dissemination. Given the multifunctional activities that have potentially important impacts on the pathogenesis of leptospirosis, it is of interest to further characterize the structure-function properties of LigB in support of future biochemical studies and the improvement of diagnostics and vaccines that are currently limited by the strain-specific lipopolysaccharide coat of the leptospires [1], [2], [3]. Thus, we have identified the repeat domains in LigB that are important for binding to fibronectin and fibrinogen. In addition, we show that leptospirosis patients make antibodies for the binding domains in LigB.

## Materials and Methods

### Assurance of ethical use of human samples

This study was conducted according to the principles expressed in the Declaration of Helsinki. Informed written consent was obtained from participants and the study was approved by the Institutional Review Board of the Research and Development Committee, VA Greater Los Angeles Healthcare System (PCC # 2008-121778).

### Recombinant proteins

The *ligB* gene from *Leptospira interrogans* serovar Copenhageni strain Fiocruz L1-130 was subcloned to produce proteins comprising ordered deletions of the LigB-specific immunoglobulin-like repeats (**Table 1**). The nomenclature for the recombinant Lig proteins is also indicated in **Table 1**. The cloning procedures have been described [15], with the addition of the PCR primers for the new proteins made available as a supplement here (**Table S1**). Repeat 11 in LigB9-11 was replaced with repeat 8 or repeat 12 by DNA polymerase-mediated amplification of the fragment for repeats 9 to 10 with the forward primer for repeat 9 and a phosphorylated reverse primer for repeat 10 lacking an *Xho* I site and any leader sequence (**Table S1**). The *Nde* I-digested DNA was then blunt-end ligated to the *Xho* I-digested DNA fragment for repeat 8 or repeat 12 that was amplified with the respective phosphorylated forward primer lacking an *Nde* I leader sequence and the respective reverse primer containing an *Xho* I site (**Table S1**). The ligation product was cloned into pET-20b(+) (Novagen, San Diego, CA). The LigB protein cloned from *L. interrogans* serovar Pomona strain LC82-25 is also described in **Table S1**. The expression of soluble recombinant protein in *Escherichia coli* BLR(DE3)pLysS (Novagen) with isopropyl-β-D-thiogalactopyranoside induction at 30°C and purification with nickel-affinity chromatography have been described [15]. Proteins were stored sterile at 4°C to avoid denaturation from freeze-thawing and were inspected for insoluble material and breakdown.

**Table 1:** Recombinant LigB proteins.

*protein* a	*amino acid coordinates* b	*mol. wt.* c
**LigB7′-12**	A631-A1125	52,310
**LigB8-12**	Q673-A1125	48,170
**LigB9-12**	L760-A1125	39,329
**LigB10-12**	K851-A1125	29,581
**LigB11-12**	T942-A1125	20,173
**LigB7′-9**	A631-L850	23,942
**LigB7′-10**	A631-A941	33,350
**LigB7′-11**	A631-A1031	42,777
**LigB9-10**	L760-A941	20,370
**LigB9-11** d	L760-A1031	29,796
**LigB9-11′**	L760-S985	28,489
**LigB9′-11**	S803-A1031	28,432
**LigB9-10-8**	L760-A941 + Q673-A759	29,210
**LigB9-10-12**	L760-A941 + T1032-A1125	29,902
**LigB10-11**	K851-A1031	20,048

a The unique repeats of LigB from *Leptospira interrogans* Copenhageni L1-130 were subcloned in pET-20b(+) at the *Nde* I/*Xho* I site, which adds an initiating methionine and an LEH_6_ tail to the Lig protein. The recombinant proteins are named for their repeats; the prime designation indicates a partial repeat.

b LigB-unique repeats are residues 582 to 1118 (full repeat 7 to repeat 12). The specific repeats are **7**: K582-V669, **7**′: A631-V669, **8**: Q673-V756, **9**: L760-I845, **10**: K851-F936, **11**: T942-V1028, and **12**: T1032-V1118. All constructs include the short 3- to 5-residue linker following the last repeat, except repeat 12, which includes seven residues into the LigB C-terminal domain (amino acids 1119 to 1890).

c includes pET-encoded amino acids.

d also cloned with N-terminal H_6_ in pET-14b at the *Nde* I/*Xho*I site, producing a protein with mol. wt. 33,174.

### Fibrin formation

Fibrin formation from purified fibrinogen was examined following a 30-min preincubation at room temperature in 100 µL of 3 µM human fibrinogen (HYPHEN BioMed, Mason, OH) with 5 µM LigB9-11 or SdrG, a fibrinogen-binding protein from *Staphylococcus epidermidis* (generous donation from Dr. Magnus Höök; [19]) in phosphate-buffered saline (PBS), pH 7.2. Clotting was initiated with 0.1 U/mL human thrombin (HYPHEN BioMed) and monitored by absorbance at 600 nm every minute for 15 min; the increase in optical density corresponded with noticeable gel formation. LigB11-12 and bovine serum albumin (BSA) were tested with no effect on fibrin formation.

### Host-protein binding by LigB proteins

LigB protein binding purified host proteins was measured with an enzyme-linked immunosorbent assay (ELISA) as previously described [15]. Purified human fibronectin from plasma (Sigma-Aldrich, St. Louis, MO) or fibroblasts (Calbiochem, San Diego, CA), the 35-kDa N-terminal domain, the 45-kDa gelatin-binding domain, and the 70-kDa N-terminal fragment of fibronectin (all from Sigma-Aldrich), fibrinogen (HYPHEN BioMed), fibrinogen fragment D (HYPHEN BioMed), and collagen type III (Sigma-Aldrich) were each immobilized in microtiter wells by overnight incubation of 1 µg in 100 µL PBS, pH 7.2, at 4°C. Non-specific binding sites were blocked with 20 mg/mL BSA in PBS, pH 7.2, before incubation with recombinant LigB in 20 mg/mL BSA for 1 h at 37°C. After washing with PBS, bound LigB was located with a monoclonal antibody for its polyhistidine tag (Novagen) and measured by the absorbance at 450 nm of the enzymatic conversion of 3,3′,5,5′-tetramethylbenzidine (TMB; Thermo Scientific, Rockford, IL) by horseradish peroxidase coupled to a goat antibody for murine IgG (Novagen). Binding affinities were estimated with the apparent K_d_ derived from the LigB concentration at half-maximal binding. The mean K_d_ values and standard deviations were calculated from multiple independent experiments and compared by one-way ANOVA. The results are presented as composite curves showing the mean and standard deviation of each point compiled from multiple experiments as indicated; where n is at least 10, the standard error is shown.

### Transformation of *Leptospira biflexa*

Saprophytic *L. biflexa* serovar Patoc strain Patoc 1, which lacks the *lig* genes, was used as a non-pathogenic surrogate to express *ligA* or *ligB* from pathogenic *L. interrogans* serovar Copenhageni strain Fiocruz L1-130 (manuscript submitted by Figueira *et al.*). Briefly, *L. biflexa* was transformed by electroporation with either the pSLe94 cloning vector carrying the spectinomycin-resistance gene or pSLe94 with *ligA* or *ligB* from *L. interrogans* inserted at the *Pvu* II site for constitutive expression under the control of the borrelial *flgB* promoter. The transformants were amplified in liquid Ellinghausen-McCullough-Johnson-Harris (EMJH) medium and plated onto EMJH agar with 40 µg/mL spectinomycin at 30°C. Isolated colonies were verified for the presence of the *lig* genes by PCR and maintained in serial passage in EMJH under selection.

For the adhesion assays, the transformed leptospires were grown in EMJH medium at 30°C under spectinomycin selection to late-log phase, when surface expression of the Lig proteins has been verified (Figueira *et al.*) and the Lig content confirmed in the present study by immunoblotting with a rabbit polyclonal antibody recognizing both LigA and LigB (**Figure S1**). Cellular binding to fibrinogen and fibrinogen fragment D was measured with an ELISA ([15], Figueira *et al.*). Briefly, microtiter wells coated with 1 µg purified human fibrinogen or fibrinogen fragment D (both from HYPHEN BioMed) and blocked with 20 mg/mL BSA were incubated with 3×10^8^ leptospires at 30°C for 1 h. Non-adherent cells were removed with washes in PBS with 5 mM MgCl_2_ and the adherent cells were fixed with 4% (w/v) formaldehyde and detected by probing with a rabbit polyclonal antibody for wild-type *L. biflexa* (MyBioSource, San Diego, CA), followed by measuring the activity of horseradish peroxidase coupled to an antibody for rabbit IgG (GE Life Sciences, Piscatawny, NJ). The average increase and standard deviation in adhesion by the *lig* transformants from at least three separate experiments are reported. In individual experiments, the mean level of adhesion and standard deviation from triplicate wells of each transformant were compared to wild-type cells by ANOVA with the Dunnett post test.

### Reactivity of leptospirosis patient antibodies with Lig proteins

Antisera were collected from laboratory-confirmed leptospirosis patients in Salvador, Brazil, during acute infection and convalescence [4]. Immunoglobulin G was prepared from pooled and individual samples with Melon G resin (Thermo Scientific) and checked for purity with denaturing polyacrylamide gel electrophoresis and Coomassie Blue staining. Protein concentrations were determined with the bicinchoninic assay. The antibodies were titrated with an ELISA against recombinant LigB7′-12, LigB9-11, and *S. epidermidis* SdrG, which were immobilized in microtiter wells by overnight incubation of 100 µL of 0.5 µM protein in PBS, pH 7.2, at 4°C. Empty space was blocked with Protein-Free Blocking Buffer (Thermo Scientific) and the bacterial proteins probed with patient antibodies in 100 µL 20 mg/mL BSA in PBS, pH 7.2, at room temperature for 1 h. The PBS-washed, binding antibodies were detected with a goat antibody for human IgG conjugated to horseradish peroxidase (Sigma-Aldrich) and measured by absorbance at 450 nm of the enzymatic conversion of TMB (Thermo Scientific).

## Results

### LigB-fibrinogen binding inhibits fibrin formation

**Figure 1:**
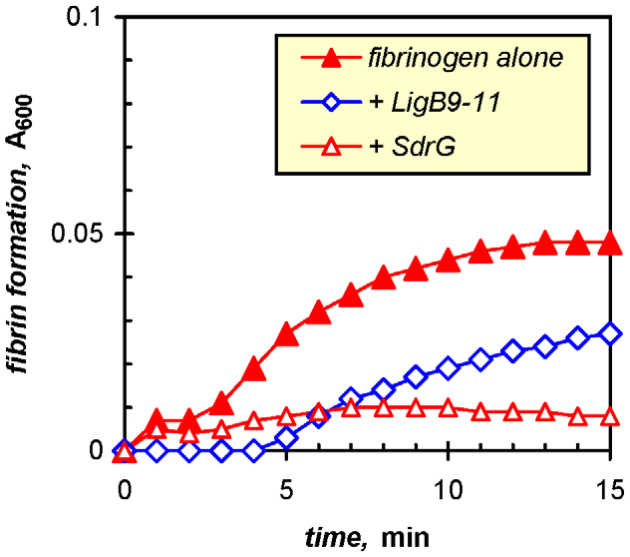
LigB binds fibrinogen and inhibits fibrin formation. Thrombin-catalyzed clotting was measured spectrophotometrically following a 30-min incubation of 3 µM human fibrinogen with PBS or 5 µM of either LigB9-11 or the *Staphylococcus epidermidis* fibrinogen-binding adhesin, SdrG.

Leptospires gaining entry in abraded or cut skin encounter an environment of increased osmolarity replete with compounds involved in wound healing and tissue repair. The osmotic change induces the expression of the Lig adhesins, which can promote the binding of the spirochetes to host proteins, such as fibronectin and fibrinogen [15], two major plasma proteins that could be lost from surrounding damaged vasculature. In freshly injured skin, hemostasis is a critical process involving clot formation from fibrinogen. In addition to enhancing leptospiral adhesion to fibrinogen in a wound (see cellular model below), LigB could modulate clotting. Its binding of fibrinogen partially inhibited thrombin-catalyzed fibrin formation, suggesting a role in countering the effects of coagulation that could impede the dissemination of leptospires from an entry site in damaged skin. In three separate determinations, LigB9-11, a recombinant LigB protein with full fibrinogen-binding activity (see below), decreased fibrin formation with purified fibrinogen by 22 to 43% (mean and standard deviation, 29±12%). A representative assay is shown in **Figure 1** along with the nearly complete suppression of clotting obtained with the *Staphylococcus epidermidis* adhesin, SdrG [19], which has two- to threefold higher avidity for fibrinogen (data not shown). Note that the significant delay in clotting caused by LigB-fibrinogen binding could also provide time for the spirochetes to enter the bloodstream and disseminate to distal tissue.

### LigB binds host proteins associated with wound healing

**Figure 2:**
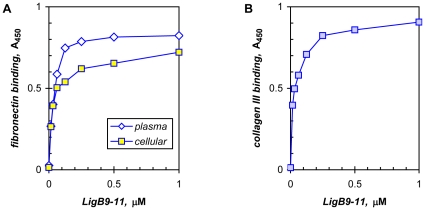
LigB binds cellular fibronectin and collagen type III. A recombinant LigB protein was tested in an ELISA for binding to **A**) human plasma and cellular fibronectin and **B**) human type III collagen.

The osmotic induction of the Lig adhesins could also enhance the attachment of the pathogen to exposed extracellular matrix containing fibronectin, collagen, laminin, and elastin, as well as to plasma proteins lost from damaged blood vessels, such as fibronectin and fibrinogen [15], [18]. Both the cellular isoform of fibronectin and collagen type III are synthesized by fibroblasts in damaged tissue, and *L. interrogans* has been shown to adhere to fibronectin made by cultured fibroblasts [20]. We have shown that LigB7′-12 (previously named LigB U1), a recombinant protein consisting of all six of the LigB-specific immunoglobulin-like repeats, binds plasma fibronectin and mediates leptospiral adhesion [15]. Here a smaller LigB protein, LigB9-11, found to retain fibronectin-binding activity with just three repeats (see below), is shown to also bind fibroblast fibronectin with similar high avidity, exhibiting apparent K_d_s of 32±15 nM (17 independent determinations) and 25±10 nM (n = 3) for plasma and cellular fibronectin, respectively (representative experiments shown in **Figure 2A**). LigB9-11 also showed high-affinity binding to collagen type III with an estimated K_d_ of 30 nM (range 10 nM for two determinations) (**Figure 2B**). Moreover, the interaction observed for LigB9-11 with type III collagen proved to be stronger than the previously measured non-saturating activities of LigB7′-12 with collagen types I and IV [15], suggesting a significant role for type III collagen binding early during infection in adhering to tissue undergoing wound healing and repair.

### Transformation with *ligA* and *ligB* enhances adhesion of nonpathogenic *L. biflexa*

**Figure 3:**
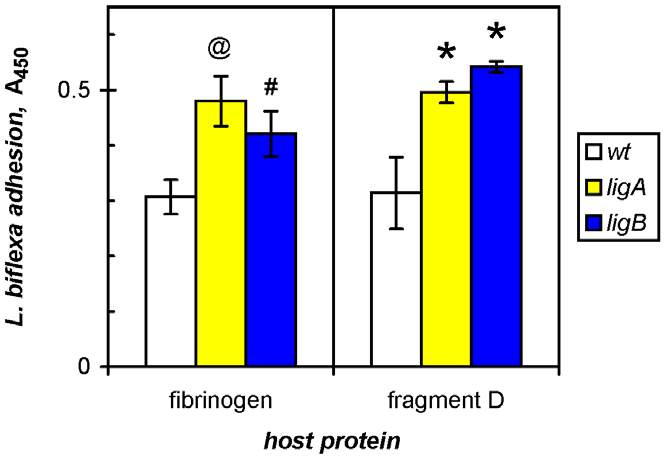
*lig* transformation of *Leptospira* saprophyte enhances adhesion to fibrinogen. Wild-type (*wt*) *L. biflexa* and cells transformed with *ligA* or *ligB* from *L. interrogans* were tested for binding to immobilized human fibrinogen or its fragment D in an ELISA. Three x 10^8^ leptospires were incubated for 1 h at 30°C and adherent cells fixed with formaldehyde and detected with a rabbit antibody to intact *L. biflexa*. A representative of multiple experiments is shown with means and standard deviations of triplicates. Statistically significant increases compared to binding by *wt* with each host protein were determined by one-way ANOVA (@, *, P<0.01; #, P<0.05).

The osmotic induction of Lig expression in pathogenic *L. interrogans* enhances spirochetal binding to immobilized fibronectin and fibrinogen in an *in vitro* model for attachment to host tissue [15]. In a direct demonstration of the role of Lig in adhesion, we have recently shown that the constitutive expression of either LigA or LigB directed by the borrelial *flgB* promoter, on the surface of nonpathogenic *L. biflexa* transformed with *ligA* or *ligB* from *L. interrogans*, enhances the adherence of the saprophyte to both cellular and plasma fibronectin (Figueira *et al.*). In accord with the high-avidity interactions observed for recombinant LigB protein with cellular as well as plasma fibronectin (**Figure 2A**), the *ligB* transformant of *L. biflexa* bound cellular and plasma fibronectin twofold more than the parental wild-type strain (Figueira *et al.*). We now show that genetic augmentation with either *ligA* or *ligB* also promoted leptospiral adhesion to fibrinogen (**Figure** **3**). The adherence of the *ligA* transformant was elevated by 55±14% (seven independent determinations) compared to either the native wild-type, nonpathogenic strain or these cells transformed with the empty pSLe94 vector. The *ligB* transformant also bound to immobilized fibrinogen 55±12% (four determinations) more than wild-type *L. biflexa*. In the representative experiment shown in **Figure 3**, *ligA* transformation increased attachment by 56% (P<0.01 compared to wild-type leptospires) and the insertion of *ligB* led to 37% more binding (P<0.05). In addition, adhesion to fragment D of fibrinogen (see below) increased by 58 and 73% for the *ligA* and *ligB* transformants, respectively, over wild-type *L. biflexa* (P<0.01; **Figure 3**).

### Fibronectin-binding sequences in LigB

We previously showed that two separate regions in fibronectin, the N-terminal and the adjoining gelatin-binding domains, are recognized by recombinant Lig proteins comprising the variable immunoglobulin-like repeats (C-terminal half of LigA, repeats 7′ to 13, and the middle portion of LigB, repeats 7′ to 12; the proteins LigA U and LigB U1, respectively [15]). A collection of recombinant terminal-deletion and other mutant LigB proteins has now been used to identify the repeat domains within LigB7′-12 that are required for the interactions with fibronectin (**Table 1**).

**Figure 4:**
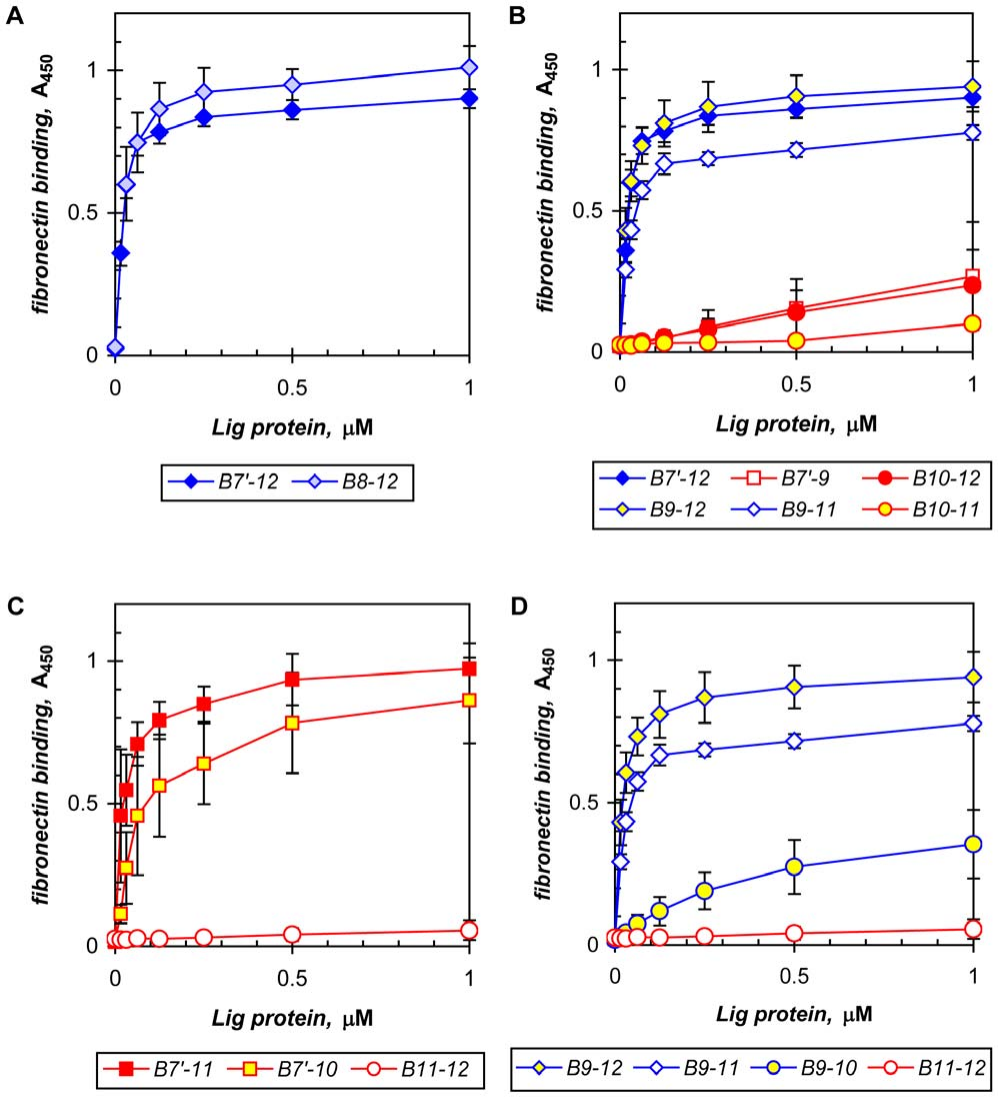
LigB repeats in fibronectin binding. Recombinant LigB proteins comprising the indicated immunoglobulin-like repeats were tested in an ELISA to identify the fibronectin-binding region. Composite binding curves with means and standard deviations (standard errors for LigB7′-12 and LigB9-11) from multiple assays depict their comparative binding avidities to demonstrate that **A**) repeat 7 is not necessary, **B**) repeat 9 is necessary along with a nexus with repeat 10, and **C**, **D**) repeats 11 and 12 do not bind directly, but are involved in the interaction with fibronectin. See text and **Table 2** for the apparent K_d_s.

Repeat 7 was not needed for LigB binding to fibronectin or its 70-kDa N-terminal fragment, which consists of the contiguous N-terminal and gelatin-binding domains (**Figure 4A** and data not shown for binding to the fibronectin domains). Omitting the half-repeat 7 in LigB8-12 maintained the same avidity observed for LigB7′-12, with apparent K_d_s of 26±3 nM (three separate determinations) and 23±8 nM (n = 18), respectively (see **Table 2** for comparative summary of binding avidities for the LigB proteins tested in this study). In contrast, LigB repeat 9 was necessary for binding fibronectin and its N-terminal fragment. Whereas LigB10-12 and LigB10-11 were weak or inactive in binding, the addition of repeat 9 to either protein reconstituted virtually full activity with K_d_s of 22±3 nM (n = 5) for LigB9-12 and 31±14 nM (n = 19) for LigB9-11 (**Figure 4B**). The N-terminal portion of repeat 9 was crucial. Deletion of the N-terminal half of repeat 9 in LigB9-11 caused complete inactivation (**Table 1**; data not shown). Another modification that tacked on a hexahistidine tag along with 15 vector-encoded residues to the amino terminus of repeat 9 in LigB9-10 and LigB9-11 also ablated fibronectin binding (**Table 1**; data not shown).

**Table 2:** Relative affinities of LigB proteins for fibronectin and fibrinogen.

	*Fibronectin*		*Fibrinogen*		
LigB	K_d_ a	%^b^	K_d_ a	%^b^	Category
7′-12	23±8	100	34±11	100	*full binding*
7′-11	27±14^c^	100	see below	-	
8-12	26±3	100	n.d.	-	
9-12	22±3	100	37±13	100	
9-11	31±14^d^	100	27±9^d^	100	
7′-11	see above	-	63 (28)	54	*moderate/low*
7′-10	77±47c, e	30	207 (165)	16	
9-10	252±30d, e	9	169±40^d^	20	
7′-9	weak	-	weak	-	*weak*
10-12	weak	-	weak	-	
10-11	inactive	-	n.d.	-	*inactive*
11-12	inactive	-	inactive	-	

a Apparent dissociation constant estimated by ELISA. Mean and standard deviation from at least three separate experiments is shown, except where the mean from two experiments is shown with the range in parentheses; n.d., not determined.

b Relative affinity compared to LigB7′-12; where 100% is shown, the affinities are statistically equivalent.

c Significant 2.9-fold enhancement by repeat 11 (P<0.01).

d Significant 8.1-fold enhancement by repeat 11 for fibronectin binding (P<0.001), and 6.3-fold stimulation for fibrinogen binding (P<0.001).

e Some dependence on repeats 7′ and 8 in the absence of repeat 11, giving a 3.3-fold effect (P<0.001).

The two most carboxy-proximal immunoglobulin-like repeats in LigB, 11 and 12, by themselves did not bind fibronectin or the 70-kDa N-terminal fragment (**Figure 4C, 4D**, and data not shown). Different preparations of purified LigB11-12 were tested and found to be non-binding. In addition, of all LigB proteins tested, only repeats 11 and 12 did not bind fibronectin. Moreover, the addition of repeat 10 to the inactive repeats in LigB10-11 and LigB10-12 supported only weak binding at best, suggesting that repeats 11 and 12 did not harbor any cryptic binding activity (**Figure 4B**). Although Lin *et al.* had located a fibronectin-binding domain in this part of LigB from *L. interrogans* serovar Pomona [21], [22], we subcloned a LigB protein consisting of repeats 11 and 12 from a Pomona strain and found it to be inactive (**Table S1**; data not shown).

As demonstrated above, repeat 9 was necessary for fibronectin binding; however, it alone was not sufficient to account for the full activity of LigB7′-12. A nexus between repeats 9 and 10 was also needed; dividing LigB7′-12 between these two repeats produced two low-activity half-molecules, LigB7′-9 and LigB10-12 (**Figure 4B**). A second requirement for repeats 9 and 10 to fully express their fibronectin-binding activity was the non-binding repeat 11. For example, LigB7′-10 displayed only moderate activity with an estimated K_d_ of 77±47 nM (n = 7) (**Figure 4C**). However, the addition of repeat 11 conferred full binding activity as shown by LigB7′-11 with K_d_ 27±14 nM (n = 4; significantly stronger than LigB7′-10, with P<0.01) (**Figure 4C**). The requirement for repeat 11 was also evident in comparing the moderate activity of LigB9-10 (K_d_ 252±30 nM, n = 5) to the tight binding by LigB9-11 and LigB9-12, both being significantly different from LigB9-10, with P<0.001 (**Figure 4D**). Thus, the sequences involved in high-affinity interaction with fibronectin are contained in LigB9-11. The enhancement mediated by repeat 11 appeared somewhat tolerant of sequence requirements. Although deletion of the carboxyl half of repeat 11 diminished the activity of LigB9-11 by 80%, as did replacing repeat 11 with repeat 8 or 12, complete inactivation did not occur (**Table 1**; data not shown). Note that in the absence of repeat 11, fibronectin binding was somewhat dependent on repeats 7′ and 8: LigB7′-10 compared to LigB9-10, with K_d_s of 77±47 nM and 252±30 nM, respectively (**Table 2**).

### Fibrinogen-binding sequences in LigB

**Figure 5:**
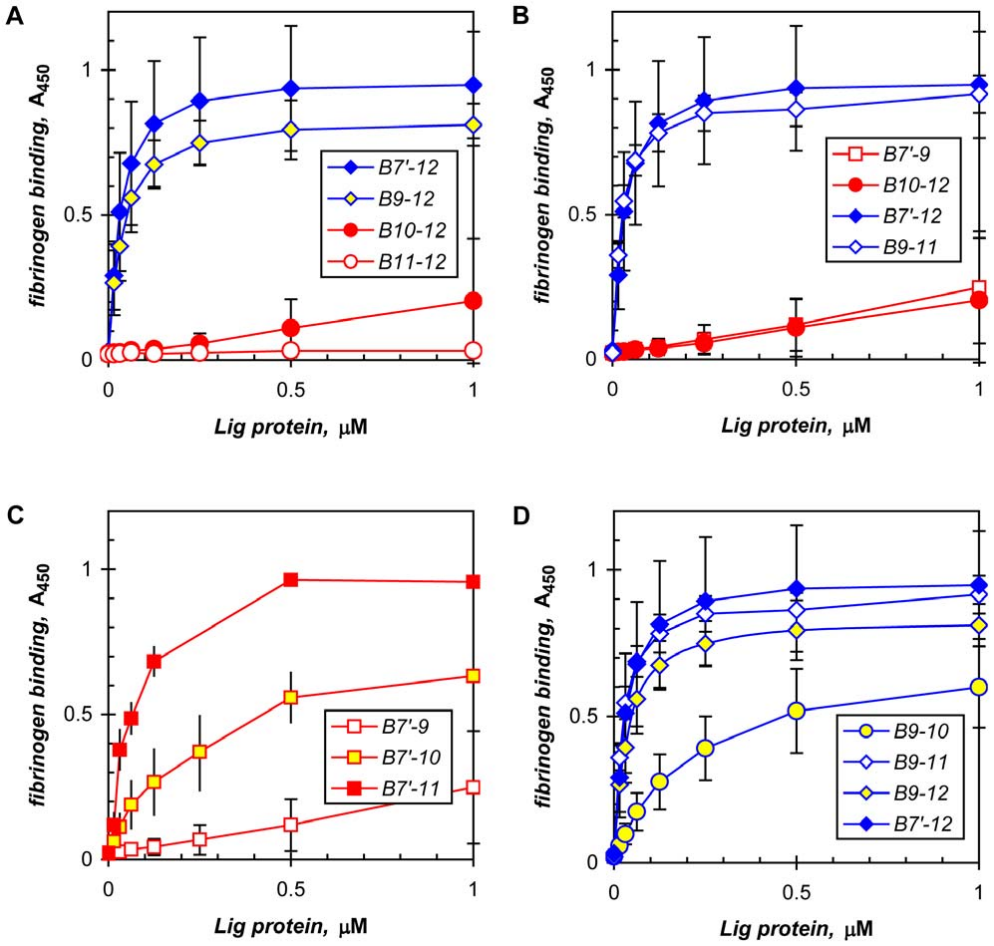
LigB repeats in fibrinogen binding. Recombinant LigB proteins comprising the indicated immunoglobulin-like repeats were tested in an ELISA to identify the fibrinogen-binding region. Composite binding curves with means and standard deviations (standard errors for LigB9-11, and ranges for LigB7′-10 and LigB7′-11) from multiple experiments show that **A**) repeat 9 is necessary and the carboxy-proximal repeats, especially 11 and 12, do not bind by themselves, **B**) a repeats 9-10 nexus is necessary and B9-11 is sufficient for full activity, **C**) low- (10) and non-binding (11) repeats enhance activity, and **D**) non-binding repeat 11 is needed for complete fibrinogen binding. See text and **Table 2** for comparative K_d_s.

In addition to binding fibronectin (**Figure 2A**, **4**), LigB7′-12 and its fibronectin-binding region identified above in repeats 9 to 11 recognized fragment D, the globular portion of human fibrinogen containing the C-terminal portions of the α-, β-, and γ-chains ([23]; **Figure 5A, 5B**). Equivalent results were obtained with both whole fibrinogen and its fragment D. Binding by LigB9-11, for example, gave statistically equivalent K_d_s of 28±14 nM (n = 4) with fibrinogen and 26±6 nM (n = 6) with fragment D; results are thus reported for either protein interchangeably. The repeats that are important for fibrinogen interaction have been mapped to the fibronectin-binding region of LigB. Thus, repeat 9 was also necessary for fibrinogen binding as shown by the marked enhancement in activity with its addition to the virtually inactive LigB10-12 (**Figure 5A**). The apparent K_d_ of 37±13 nM (n = 3) for LigB9-12 matched that for LigB7′-12, 34±11 nM (n = 7) (see **Table 2** for a comparison of binding affinities for the LigB proteins tested). As with fibronectin, repeats 11 and 12 by themselves did not bind fibrinogen (**Figure 5A**). Thus, repeats 9 and 10 were also important for fibrinogen binding. Here an association between the two repeats was also required; separating them as in LigB7′-9 and LigB10-12 significantly weakened the activity observed with intact LigB7′-12 (**Figure 5B**). LigB9-11 containing the two repeats and constituting the fibronectin-binding region of LigB was also sufficient to support full fibrinogen-binding activity, with a K_d_ of 27±9 nM (n = 10) being statistically the same as that for LigB7′-12 (**Figure 5B**). In addition, the obligatory nexus between repeats 9 and 10 was evident in the significant increases in the activities of LigB9-12 v. LigB10-12 (**Figure 5A**) and LigB7′-10 v. LigB7′-9 (**Figure 5C**). As with fibronectin binding, the N-terminal portion of repeat 9 was particularly sensitive to alterations. The addition of non-Lig sequence, such as 15 vector-encoded residues plus a His tag, to its amino terminus rendered LigB9-11 virtually inactive with less than 10% residual activity. Deletion of the amino half also eliminated fibrinogen binding (**Table 1**; data not shown).

The non-binding repeat 11 also served as an enhancer in the interaction with fibrinogen. Its addition to LigB7′-10 (**Figure 5C**) and LigB9-10 (**Figure 5D**), two proteins with moderate activity, increased fibrinogen binding substantially by several fold (**Table 2**). For example, LigB9-11 had sixfold higher affinity for fibrinogen than LigB9-10: K_d_ 27±9 nM (n = 10) v. 169±40 nM (n = 4), respectively (P<0.001 by ANOVA; **Figure 5D**). The full activity obtained with LigB9-11, which was equivalent to the activities of LigB9-12 and LigB7′-12, clearly showed that repeat 11 alone was sufficient to enhance fibrinogen binding by LigB9-10 (**Figure 5D**). Removal of the carboxyl half of repeat 11 lowered the activity of LigB9-11 by 50%, suggesting that the intact non-binding repeat was necessary to support optimal fibrinogen binding (**Table 1**; data not shown).

### Host antibody responses to ligand-binding domain in LigB

**Figure 6:**
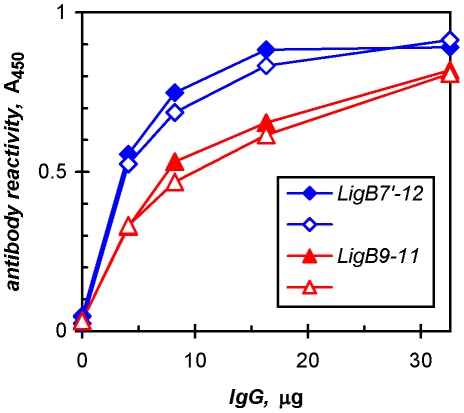
Leptospirosis patient antibodies recognize fibronectin/fibrinogen-binding region of LigB. Immunoglobulins purified from a pool of antisera of patients with acute infection (closed symbol) and from a convalescent patient (open symbol) were titrated against LigB proteins in an ELISA.

The LigB repeats involved in host-protein binding presumably have some degree of exposure on the leptospiral surface for their interaction with cognate ligands. In order to determine whether this functional accessibility would also render the repeats vulnerable to host immune responses, the antigenicities of the recombinant LigB proteins were compared. Immunoglobulin G purified from patient antisera collected during acute leptospirosis infection and during convalescence recognized epitopes in LigB7′-12 as well as in LigB9-11 (**Figure 6**), reflecting the expression of LigB by the infecting leptospires and perhaps its surface-available fibronectin/fibrinogen-binding domain. The *S. epidermidis* fibrinogen-binding protein, SdrG, was used as a negative control antigen without reactivity (data not shown).

## Discussion

### LigB expression and binding profiles suggest an early role in cutaneous infection

The risk of leptospirosis commonly occurs in impoverished urban habitats where seasonal flood waters become contaminated by rodent urine. However, flowing rivers and lake water contaminated with presumably much lower concentrations of leptospires than in urban floods also present significant risks. Indeed, the infection of humans and animals in open water is evidence of the effective mechanisms used by pathogenic *Leptospira* spp. in the productive initial contact with hosts [5], [6], [7]. Whereas it seems clear that damaged skin is infected during urban flooding or agrarian work in contaminated soil, it is less clear how mucous membrane is infected from exposure such as in water sports. The externally exposed mucosa of the eye, nose, mouth, and respiratory tract is a formidable barrier. Indeed, both dermis and mucosa have complex immune systems [24], [25]. In addition to containing immune cells, such as dendritic cells, mucous membranes secrete a protective layer of mucus that harbors antibodies, primarily IgA [26], [27], [28]. In a previous study of the guinea pig model of leptospirosis that compared different routes of infection, the LD_50_ for subcutaneous injection was twofold higher than with an intraperitoneal bolus, and conjunctival inoculation was yet another three orders of magnitude higher than in subcutaneous infection [29], attesting to the effectiveness of the mucosal barrier. The results from the present study suggest that there are specific mechanisms mediated by LigB that promote the transmission of leptospirosis *via* damaged skin.

Healthy skin and its appendages, such as hair and fur, serve as an effective barrier to pathogens. However, damaged skin provides leptospires not only an opportunity for physical access into a host, but also exposure to metabolic processes that are activated in a wound. At the time of initial contact with the leptospires, the injury will be at one of several stages of healing and repair. Some of these processes may be conducive to the successful initiation of infection by a pathogen. One of the very first to occur in a fresh wound is hemostasis involving the plasma protein, fibrinogen, in the damaged vasculature [8], [9], [10], [11]. The subsequent repair process includes fibroblasts recruited to the site that lay down a temporary extracellular matrix containing mostly cellular fibronectin and type III collagen [8], [9], [10], [11], [12], [13]. Our results suggest that invading leptospires could adapt to this environment. In addition, the inflammatory responses that accompany tissue damage likely have effects on leptospires that are yet to be understood.

Furthermore, other mammalian stimuli to which pathogenic leptospires respond include physiological osmolarity [16], [17] and temperature [30], [31], which have been shown to rapidly induce the expression of LigA and LigB, making these proteins potential mediators of interactions with the host especially at the onset of an infection. The transient induction of Lig expression by host body temperature [30] and the unknown persistence of osmotic induction suggest that the primary functions of the Lig proteins are assigned to early infection. In addition, elastin binding has been described for LigB [18], suggesting a function early in cutaneous infection involving elastin-rich skin and damaged vasculature. Thus, our hypothesis is that the Lig proteins, especially LigB, which we previously showed to bind the human plasma proteins, fibronectin and fibrinogen, with high avidity [15], help to mediate successful infections in cutaneous wounds. In support of this, we show that LigB binds fibrinogen and inhibits fibrin formation (**Figure 1**), and that LigB binds fibroblast-derived fibronectin and type III collagen with nanomolar avidity (**Figure 2**).

The inhibitory effect of LigB on fibrin formation (**Figure 1**) would occur during leptospiral contact with a fresh wound, since fibrinogen-mediated hemostasis occurs at the onset of an injury. However, the disruption of fibrin formation could adversely affect processes beyond hemostasis that involve interactions with fibrin and the fibronectin associated with it in a clot, such as wound stabilization, platelet function, phagocytosis, tissue repair, and angiogenesis [8], [9], [10], [11], [32], [33]. Thus, their impairment could conceivably promote the initiation of an infection and facilitate further infection and dissemination. The moderate level of inhibition by LigB compared to the complete prevention of clotting by the *S. epidermidis* fibrinogen-binding protein, SdrG (**Figure 1**; [19]), suggests that the primary function of LigB is in adhesion. Another fibrinogen-binding protein, ClfA from *S. aureus*, also disrupts fibrin formation [34]. It is of note that immunoglobulin-like repeats in these three fibrinogen-binding proteins affect clotting by interacting with different parts of fibrinogen. LigB binds fragment D (specific chain unidentified), SdrG binds an N-terminal site in the β-chain in fragment E [19], and ClfA binds a C-terminal site in the γ-chain in fragment D [35], [36], [37].

The plausibility of LigB having a role during early infection in the skin raises the issue of the suitability of animal models of leptospirosis that use experimental methods such as intraperitoneal injection instead of natural routes of infection. It is possible that the former might systematically preclude the detection of mechanisms required for infection at a natural barrier or at low dose. This would apply in the case of gene knock-out models that seek to identify virulence factors, e.g., in the *ligB* knock-out study that found no decrease in the infectivity of hamsters with the *ligB*-null mutant compared to wild-type leptospires [38]. One interpretation is functional redundancy for an important step carried out by LigB. For example, LigA also binds host proteins that LigB binds [15]. Alternatively, the experimental conditions, such as the route and dosage of infection, may not have been suitable to examine the requirements for low-dose, natural infection, e.g., in contaminated river water.

### Saprophyte transformation as a cellular model of pathogenic function

Fibrinogen binding by LigB could serve several purposes in establishing an infection in damaged skin. In addition to disrupting hemostasis, LigB binding could mediate leptospiral adhesion to tissue via fibrinogen lost from damaged vasculature and deposited in the extracellular space. Conversely, leptospires could enter the bloodstream through damaged vessels, followed by Lig binding to circulating fibrinogen as a vehicle for dissemination to distal tissue. Our results with purified recombinant LigB proteins support these potential roles for Lig-mediated leptospiral attachment to fibrinogen.

Moreover, by applying a novel cellular model created to test for virulence factors, we have demonstrated the potential role of cellular LigB (and LigA) in leptospiral adhesion (Figueira *et al.*). In contrast to the Lig expression and resulting enhanced adhesion observed with *L. interrogans* [15], [16], [17], osmotic induction is not necessary for surrogate expression, which is constitutive and significantly increases leptospiral binding to two homeostatic proteins found in damaged skin, cellular fibronectin (Figueira *et al.*) as well as fibrinogen (**Figure 3**).

### LigB repeats 9 to 11 form the binding domain for fibronectin and fibrinogen

The immunoglobulin-like repeats in LigB are involved in binding elastin in addition to fibronectin and fibrinogen as well as laminin and collagen types I, III, and IV ([15], [18], [21]; **Figure 2B**). We have found the binding regions for both fibronectin and fibrinogen in LigB to overlap within repeats 9 to 11, with repeats 9 and 10 binding directly and repeat 11 acting as an enhancer (**Table 2**). With little variation, the same LigB proteins exhibit full activity with similar avidity for both host proteins (**Table 2**). In the moderate-affinity group, the LigB proteins contain the repeats 9/10 interface, but lack repeat 11, with the exception of LigB7′-11 in fibrinogen binding being at the high end of this category. The promotion of the proteins in the moderate-binding group to full activity entails three- to eightfold enhancement by repeat 11 for fibronectin binding and three- to sixfold stimulation in fibrinogen binding (**Table 2**). Although we have not determined the sequences required for collagen binding, LigB9-11 also binds type III collagen (**Figure 2B**).

Immunoglobulin-like repeats in other bacterial adhesins are organized in bipartite domains to bind different host proteins, such as the N2 and N3 subdomains in ClfA and ClfB from *S. aureus* and SdrG from *S. epidermidis* that bind fibrinogen, and the N1 and N2 subdomains in CNA from *S. aureus* that bind collagen [37], [39], [40], [41], [42]. The *S. aureus* fibronectin-binding adhesin, FnBPA, also binds fibrinogen and elastin [43]. Its N-terminal A region has a three-dimensional structure similar to the fibrinogen-binding N2-N3 domains in ClfA and SdrG; in FnBPA, the A region binds both fibrinogen and elastin [43]. As discussed above, the common domains in these adhesins bind different sites in fibrinogen. Interestingly, although similar, ClfA binds the γ-chain and ClfB the α-chain [35], [36], [37], [44]. Thus, the same or similar sequences or conformations of immunoglobulin-like repeats in bacterial proteins can interact with heterogeneous host ligands. It remains to be determined whether LigB binds fibrinogen and other proteins with its immunoglobulin-like repeats in a mechanism similar to those of the staphylococcal adhesins. However, it is likely that the ability of leptospires to gain entry to a host is substantially enhanced by the ability of the LigB immunoglobulin-like repeats to bind multiple homeostatic proteins that are abundant in damaged skin undergoing repair.

Although repeats 7 and 8 are not necessary for binding, they are not inactive in binding (data not shown). In contrast, repeats 11 and 12 alone do not have any direct binding activity (**Figure 4C, 4D**, **5A**; **Table 2**). However, in their native context with other repeats, such as 9 and 10, they may acquire binding activity that mediates the enhancement obtained with repeat 11. Given that the immunoglobulin-like repeats are highly similar [45], it is possible that the nature of their activity, whether it is binding directly to fibronectin or fibrinogen or imparting conformational changes that modulate binding, depends on their interaction with other repeats. The evidence in support of interactions between LigB repeats includes the requirements for the apposition of repeats 9 and 10 (**Figure** **4B**, **5B**; **Table 2**) and the inclusion of the otherwise non-binding repeat 11 for LigB9-10 to express its full binding activity (**Figure** **4D**, **5D**; **Table 2**). Both requirements are reminescent of the mechanisms used by the staphylococcal adhesins discussed above. The N2-N3 and N1-N2 domains first bind fibrinogen and collagen, respectively, followed by the interaction of adjoining downstream sequences with the binding domains [37], [41], [42]. In LigB, the essential nexus of repeats 9 and 10 is evident in comparing the full fibronectin- and fibrinogen-binding activities of LigB7′-12 to the low activities of LigB7′-9 and LigB10-12 (**Figure 4B**, **5B**; **Table 2**). It appears that the putative bipartite binding domain can be formed by intermolecular interaction; an equimolar mixture of LigB8-9 and LigB10-11, each weakly binding, yielded fibronectin-binding activity similar to LigB9-10 (data not shown). In contrast, a similar mixture of non-binding LigB11-12 plus moderate-binding LigB9-10 did not lead to an enhancement (data not shown), suggesting that repeat 11 modulates fibronectin binding by an intramolecular effect on the conformation of LigB. The disruption of productive fibronectin binding by the replacement of repeat 11 in LigB9-11 with either repeat 8 or repeat 12 is possibly due to an adverse effect on the active conformation of repeats 9 and 10. Indeed, the addition of a non-Lig sequence, such as a 21-residue vector-encoded His-tag cassette, to its amino terminus inactivated LigB9-11, possibly due to the placement of a hydrophilic region that does not occur in the naturally adjoining repeat 8 and that can perturb the interaction of repeat 9 with repeats 10 and 11.

Other studies with LigB from *L. interrogans* serovar Pomona found that LigBCen2, a recombinant protein comprising a partial repeat 11, repeat 12, and a 47-amino acid extension into the non-repeat C-terminal domain of LigB, could account for binding the N-terminal domains of fibronectin [21], [22]. The interaction with the gelatin-binding domain of fibronectin showed micromolar avidity with a K_d_ of 1.9 µM, although no binding was observed with individual repeats 10 and 11 (repeat 12 not shown, [46]). In general, the recombinant LigB proteins from serovar Pomona showed lower micromolar avidity in fibronectin binding compared to the nanomolar avidity reported here for the proteins from serovar Copenhageni ([46]; **Table 2**). Given our finding of the disruptive effect of inserting non-Lig sequence to the amino terminus of LigB9-11, some of the differences between the results with LigB from *L. interrogans* serovar Copenhageni and those reported for serovar Pomona may be due to the addition of non-Lig N-terminal sequences to the latter protein, which included either the glutathione-S-transferase protein or a His tag [46]. Nevertheless, there is some general agreement, such as the weak or absent binding activity of repeats 10 to 12. Moreover, LigB from both serovars exhibits the enhancement in fibronectin binding by C-proximal non-binding repeats. The 30-fold increase by repeat 12 in the Pomona protein appears to be mediated by a conformational change that possibly involves inter-repeat interaction [46]. In the Copenhageni protein, repeat 11 stimulates fibronectin binding three- to eightfold (**Table 2**). There is also a similar enhancement (three- to sixfold) for fibrinogen binding by LigB from Copenhageni (**Table 2**). Interestingly, LigA did not inherit repeats 11 and 12 from its ancestor, LigB [45], and shows weaker binding activity compared to LigB [15].

Taken together, the results indicate that there are multiple binding sites in LigB for fibronectin and fibrinogen, which may present more options in designing new diagnostics and therapeutics for leptospirosis. The activity of immunoglobulin-like repeats in bacterial proteins is not restricted to the binding of extracellular host proteins, but also includes the interaction with receptors on host cells, such as β_1_ integrins for invasin of pathogenic *Yersinia* spp. and Tir for intimin of pathogenic *Escherichia coli* [47], [48]. Furthermore, although they are not considered to be immunoglobulin-like repeats, the eleven tandem repeats in FnBPA from *S. aureus* that are downstream of the fibrinogen- and elastin-binding A region individually bind fibronectin with varying affinities [49]. Thus, it is clear that bacterial pathogens have adopted variations on the successful theme of using tandem repeats of structures such as the immunoglobulin fold to interact with host proteins, thereby promoting productive infections.

### Immune response in leptospirosis to fibronectin/fibrinogen-binding domain

The antigenicity of the binding domain in LigB appears to account for a large part of the human immune response to LigB (**Figure 6**), suggesting that LigB function is vulnerable to disruption during infection. However, although an antibody response is raised to LigB during acute infection, it would not occur prior to the early activities proposed for the newly induced leptospiral protein in a cutaneous wound. In addition, the binding to repair proteins, such as fibroblastic fibronectin, type III collagen, and elastin, following the initial hemostasis could occur away from circulating antibodies raised either in a previous infection or by vaccination. Therefore, the favored functional niche of LigB may well be early in infection, particularly during cutaneous entry. It is thus possible that whereas the conservation of LigB in pathogenic strains [45] and the presence of LigB antibodies in both the acute and convalescent phases of leptospirosis support the feasibility of antibody-based diagnosis [50], LigB vaccines may not be effective against the initial phase of transmission mediated by the early activities of LigB. Once it moves into the bloodstream to disseminate, the pathogen would presumably become susceptible to antibodies. Immunization with LigA protects hamsters from dying after non-cutaneous, direct systemic infection *via* intraperitoneal inoculation [51]. However, vaccination with a protein similar to LigB7′-12 does not [51], perhaps affirming the role of LigB early in the transmission of leptospirosis.

## Supporting Information

Figure S1 **Lig expression in** ***L. biflexa*** **transformed with** ***lig*** **genes from** ***L. interrogans*** **.** LigA and LigB expression was measured with an immunoblot of extracts from 1×10^8^ leptospires, using a rabbit polyclonal antibody that recognizes both proteins. **Lane 1**, Osmotic induction of Lig expression in *L. interrogans* Copenhageni Fiocruz L1-130 grown overnight in EMJH with 120 mM NaCl. **Lane 2**, *L. biflexa* Patoc parental strain grown in serum-free EMJH. **Lane 3**, *L. biflexa ligA* transformant. **Lane 4**, *L. biflexa ligB* transformant. The positions of LigA (*A*) and LigB (*B*) are marked; the smaller protein in the *ligB* transformant that also occurs partially obscured and slightly larger than LigA in *L. interrogans* is a breakdown product of LigB also detectable with a LigB-specific antibody.
(PDF)

Table S1 **Primers for cloning LigB proteins.**
(PDF)
