# Supplementary figures and images for: The Multifunctional LigB Adhesin Binds Homeostatic Proteins with Potential Roles in Cutaneous Infection by Pathogenic Leptospira interrogans
Source: PLoS One. 2011 Feb 9;6(2):e16879. doi: 10.1371/journal.pone.0016879 (PMC3036719; doi:10.1371/journal.pone.0016879)

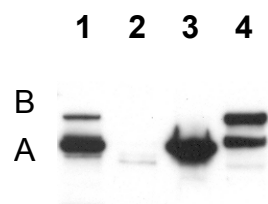

Supplement: Figure S1 — Lig expression in L. biflexa transformed with lig genes from L. interrogans . LigA and LigB expression was measured with an immunoblot of extracts from 1×10^8^ leptospires, using a rabbit polyclonal antibody that recognizes both proteins. Lane 1, Osmotic induction of Lig expression in L. interrogans Copenhageni Fiocruz L1-130 grown overnight in EMJH with 120 mM NaCl. Lane 2, L. biflexa Patoc parental strain grown in serum-free EMJH. Lane 3, L. biflexa ligA transformant. Lane 4, L. biflexa ligB transformant. The positions of LigA (A) and LigB (B) are marked; the smaller protein in the ligB transformant that also occurs partially obscured and slightly larger than LigA in L. interrogans is a breakdown product of LigB also detectable with a LigB-specific antibody. (PDF) [file pone.0016879.s001.pdf]
