# Supplementary material for: The Multifunctional LigB Adhesin Binds Homeostatic Proteins with Potential Roles in Cutaneous Infection by Pathogenic Leptospira interrogans
Source: PLoS One. 2011 Feb 9;6(2):e16879. doi: 10.1371/journal.pone.0016879 (PMC3036719; doi:10.1371/journal.pone.0016879)
Supplement: Table S1 — Primers for cloning LigB proteins. (PDF) [file pone.0016879.s002.pdf]

Table S1. Primers for cloning LigB proteins.

Forward primers [a]	
7'	TATAAACATATGGCTGAAATTAAAAATACCAGTGGAAG
8	TATTTACATATGCAATTGATTTCCATTGCCGTA
P-8[b]	CAATTGATTTCCATTGCCGTA
9	ATACTCCATATGCTTCTTACTTCTATCGAGATAACACCG
9'[c]	CTGCTTCATATGTCTTCCGATCCATCTAAGATTG
10	ACCGTACATATGAAACTGAAAAGTATAACTATCAGTCCTTCC
11	ATACATATGACGTTAGATTCCATTAAAATCAATCCAGT
11'$_{Pom}$[d]	GATCGACATATGACTGCGACTTACAATTCCATC
P-12[b]	ACCCTTTCTTCGATTTCAATATCTCCTATCAA

Reverse primers [e]	
8	TAAAATCTCGAGTGCAGGAGTGACATTCAAAAC
9	TTTAATCTCGAGTAAGTCAGTGACTGTAATCGGAATTG
10	ATTTTACTCGAGGGCAGCACTTACATTAAAATTTATTTTA
P-10[b]	GGCAGCACTTACATTAAAATTTATTTTATTACTGCTTATAG
11	ATATTTCTCGAGTGCTGCGCTGACCGTTATG
11'[f]	CTGTTACTCGAGACTGGACCAGGTAACTGAATC
12	ATTTAACTCGAGAGCTATCGTGTCCGTTTTGTTTAC
CTD$_{Pom}$[g]	CTATTTCTCGAGTTAATTGGAACTATTAATTATTTTGTAATTGG

[a]Forward primers are named for the respective LigB repeats as in **Table 1** and displayed 5' to 3' with leaders containing an *Nde* I restriction endonuclease site that is underlined. See **Table 1** for the initial amino acid of each LigB repeat from *L. interrogans* serovar Copenhageni strain Fiocruz L1-130.

[b]5'-phosphorylated for blunt-end ligation to 3'-end of linker-encoding sequence following repeat 10.

[c]N-terminal-deleted repeat 9 starting at S803.

[d]N-terminal-deleted repeat 11 from *L. interrogans* serovar Pomona starting at T1014.

[e]Reverse primers contain an *Xho* I site that is underlined. See **Table 1** for the last residue of the linker sequence that follows each repeat except for repeat 12.

[f]C-terminal-deleted repeat 11 ending at S985.

[g]First 45 amino acids of the C-terminal domain in the Pomona strain ending at N1165.
